# Bicomponent PLA Nanofiber Nonwovens as Highly Efficient Filtration Media for Particulate Pollutants and Pathogens

**DOI:** 10.3390/membranes11110819

**Published:** 2021-10-27

**Authors:** Danyang Gao, Renhai Zhao, Xue Yang, Fuxing Chen, Xin Ning

**Affiliations:** Industrial Research Institute of Nonwovens & Technical Textiles, College of Textiles & Clothing, Shandong Center for Engineered Nonwovens, Qingdao University, Qingdao 266071, China; gdykdy@163.com (D.G.); 2017021352@qdu.edu.cn (X.Y.)

**Keywords:** side-by-side bicomponent fiber, electrospinning, stereo-complexation, AlCl_3_, filtration

## Abstract

Herein, a novel form of bicomponent nanofiber membrane containing stereo-complex polylactic acid (SC-PLA) was successfully produced by the side-by-side electrospinning of Poly (L-lactic acid) (PLLA) and Poly (D-lactic acid) (PDLA). We demonstrate that through these environmentally sustainable materials, highly efficient nanofiber assemblies for filtration can be constructed at very low basis weight. The physical and morphological structure, crystalline structure, hydrophobicity, porous structure, and filtration performance of the fibrous membranes were thoroughly characterized. It was shown that the fabricated polylactic acid (PLA) side-by-side fiber membrane had the advantages of excellent hydrophobicity, small average pore size, high porosity, high filtration efficiency, low pressure drop as well as superior air permeability. At the very low basis weight of 1.1 g/m^2^, the filtration efficiency and pressure drop of the prepared side-by-side membrane reached 96.2% and 30 Pa, respectively. Overall, this biomass-based, biodegradable filtration material has the potential to replace the fossil fuel-based polypropylene commercial meltblown materials for the design and development in filtration, separation, biomedical, personal protection and other fields.

## 1. Introduction

Currently, the COVID-19 pandemic and the prevalence of other infectious diseases have brought a heavy impact on humans, not only in terms of human health but also in affecting national stability and global economic development [1]. Nowadays, the number of infection and death cases is still rising, causing incessant attention all over the world [2,3]. Studies have shown that several ways of transmission for SARS-CoV-2 include droplet transmission, body fluid transmission, aerosol transmission, and contact with contaminated surfaces [4,5]. In addition, microorganisms such as bacteria and viruses in the air can attach to particulates, which gradually enter the human organs through the respiratory system, leading to cardiovascular and respiratory diseases [6,7]. Accordingly, in addition to using diagnostic tools to screen infected people and isolate them to prevent further spread of the virus, people are encouraged to wear suitable protective equipment, such as an N95 face mask, which has proved to be the most effective infection control approach [8]. Current masks almost exclusively use polypropylene-based meltblown materials as their functional filtration media, while which can lead to undesirable effects such as environmental disposal. Therefore, the study of a non-petroleum-based filter material with high efficiency, low pressure drops and excellent air permeability is very beneficial for the development in the fields of personal protection, biomedicine and filtration.

The filtration performance of fiber-based filters is closely related to the fiber diameter distribution and porous structures thus constructed. Due to intrinsic bigger diameters, large pore size, and low porosity, the untreated meltblown and spunbond fibrous membrane present unsatisfied filtration efficiency to small aerosol particles [9]. In comparison, the nanofiber membrane fabricated by electrospinning technology has superior characteristics of thin diameter, large specific surface area, interconnected pore structure, and high porosity, which are conducive to high interception and capture of tiny aerosol particles [10,11,12]. Under unremitting researches, a large amount of high molecular polymer materials, such as PVDF, PSA, PAN, and PMIA, have been successfully fabricated by the electrospinning method [13,14,15,16]. Ding et al. reported an integrated microfiber, nanofiber and nanonets filter by a combination of three layers with different fiber diameters in sequence [17]. Such multi-modal fiber size distribution is shown to be functionally superior. Interestingly, in a previous research work on side-by-side electrospinning of PLA, our team has shown that it is a promising one-step method to produce interlaced nanofibers with bimodal diameter distribution [18], which indicates that randomly arranged single and side-by-side nanofibers can be manufactured simultaneously to construct a stochastic structure for filtration. 

Polylactic acid (PLA) is a class of green semi-crystalline polyester derived from renewable resources, having biodegradability and biocompatibility, which presents more advantages than non-biodegradable petroleum-based polymers in environmentally friendly applications [5,19,20,21]. However, PLA possesses the disadvantage of the slow crystallization rate, resulting in brittleness in their mechanical properties [22]. Interestingly, the formation of stereo-complex polylactic acid (SC-PLA) between the enantiomers of PLLA and PDLA is a method for enhancing such mechanical properties [23]. It was firstly reported in 1987 that the stereo-complexion structure in which the molecular chains of two enantiomeric components were arranged side by side to construct a paired double helix chain by Ikada and coworkers [24]. The intermolecular crystallization will increase the molecular chain entanglement, and the stereo-complexation will form finer microcrystalline structure, which becomes the cross-linking point between the molecular chains. Therefore, the molecular chain entanglement points with higher density will encourage the enhanced thermal and mechanical properties of PLA [25]. Zhao et al. reported the first attempt to obtain a continuous nano-crystalline sandwich structure fabricated through the mutual diffusion of PLLA and PDLA molecules in the side-by-side fiber electrospinning method, which could be expanded to the micron level in commercial development [18]. In recent years, the researches on the SC-PLA have achieved significant progress. Except for solution and melt blending methods, it has also been reported that the supercritical fluid method and solid-state copolymerization method are also new preparation ways to synthesize SC-PLA [26,27]. Nevertheless, more efforts should be dedicated to further enhance the synthesis of SC-PLA structure as well as discussing their web structures and potential applications.

In this study, we focused on the optimized processes of SC-PLA crystallization in the bicomponent PLLA and PDLA side-by-side nanofiber membrane. In particular, AlCl_3_ salt was added to the PDLA component side to increase the attraction of opposite charges in an electrostatic field. As a consequence, two enantiomeric components got closer and contacted better with each other, thereby promoting the mutual diffusion between molecules to form more stereo-complex crystals. Besides, different contents of AlCl_3_ were adjusted to regulate the morphology of PDLA fibers and contribute to the booming progress of the side-by-side electrospinning process. Furthermore, we investigated the advanced pore structure, filtration efficiency, pressure drop, air permeability, and hydrophobicity of side-by-side fibers. Benefiting from the special modified crystals and morphology structure conformation, side-by-side nanofibers showed a more obvious filtration application prospect than single-spinning PLA fibers. In short, this enhanced bicomponent PLLA and PDLA side-by-side nanofiber membrane with an effective one-step developed crystal and stochastic hierarchical structure is a promising candidate in the fields of filtration, biomedicine, and personal protection.

## 2. Materials and Methods

### 2.1. Materials

Poly (L-lactic acid) (PLLA, Mw = 50,000 g/mol) was purchased from Nature Works (Minnetonka, MN, USA); Poly (D-lactic acid) (PDLA, Mw = 120,000 g/mol) was supplied by Changchun Institute of Applied Chemistry Chinese Academy of Sciences (Changchun, China); *N*,*N*-dimethylformamide (DMF, 98%), dichloromethane (DCM) and Aluminum Chloride Hexahydrate (AlCl_3_·6H_2_O, Mw = 241.43, AR) were all purchased from Sinopharm Chemical Reagent Co., Ltd. (Shanghai, China). All chemical reagents were of analytical grade and used without further purification except PLLA and PDLA in an industrial grade.

### 2.2. Preparation of PLLA and PDLA Solution

PLLA and PDLA particles were dissolved in the 10 g mixed DCM and DMF solvents (DCM: DMF = 8:2, m/m) to prepare 6 wt% PLLA and 8 wt% PDLA solutions, respectively. PDLA + n wt% AlCl_3_ solution was obtained by adding different contents (0.2 wt%, 0.5 wt%, 1 wt%, 2 wt%, 4 wt%) of AlCl_3_ particles into the PDLA solution, where n was 0.2, 0.5, 1, 2 and 4, accordingly. All the above solutions were stirred fully on the magnetic stirrer at room temperature for 24 h, obtaining the uniform and transparent spinning solution. In addition, each solution needed to stand for 2 h to eliminate air bubbles before electrospinning so as not to affect the electrospinning process.

### 2.3. PLA Side-by-Side Bicomponent Electrospinning Progress 

**Figure 1:**
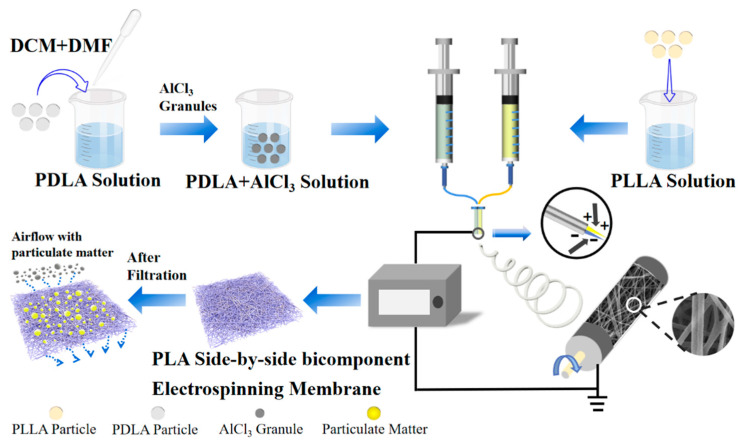
Schematic diagram of side-by-side bicomponent electrospinning nanofibers prepared and membrane surface condition before and after filtration.

The schematic diagram of the electrospinning progress and the typical morphology of the side-by-side bicomponent fiber are shown in Figure 1. High-voltage direct current supply (DW-P503-1ACF0, Dongwen High Voltage Power Supply Co., Ltd., Tianjin, China), double-channel propulsion pump (LSP02-1B, Baoding Lange Constant Flow Pump Co., Ltd., Baoding, China), and the roller receiver device together constituted the electrospinning experimental device. The configured PLLA solution and PDLA solution were loaded into two 10 mL syringes separately, which were placed on the propulsion pump. The inner and outer diameters of the needle were 0.39 and 0.63 mm. The two needles were arranged side-by-side and fixed together with hot melted glue before the catheter was connected between the needles and the syringes. In addition, a sleeve was added outside the tip of the needles, which could weaken the electrostatic repulsion between two components and reduce the phenomenon of filament separation during the spinning process. This was beneficial to the formation of more side-by-side fibers.

After pre-experimental adjustment of multiple spinning parameters, the final experimental conditions adopted were as follows: a voltage of 25 kV, the propulsion speed of 1 mL/h, the spinning distance of 17 cm, the roller receiver rotation speed of 400 r/min, the temperature at 25 ± 2 °C, and relative humidity at 35 ± 2%, respectively. Under the same conditions, PLLA, PDLA, PDLA+ n wt% AlCl_3_ fiber membranes were also prepared in a single-spinning form as the control groups, which were compared with the side-by-side bicomponent fiber membrane in numerous performances. The above process parameters were the best results after continuous improvements. The obtained bicomponent fiber membrane not only had a higher proportion of side-by-side fibers, but also had a better fiber uniformity. Besides, all electrospinning fiber membranes were stored in an oven at 60 °C for 4 h to remove the residual solvent and obtain the dried samples.

### 2.4. Characterization

Scanning electron microscopy (SEM, Phenom Pro SEM, Hitzacker, Germany) was used to observe the morphology and structure of the fibers. Nano Measure and Image J software were operated to measure the diameters of 100 randomly arranged fibers to perform diameter distribution statistics. Additionally, for side-by-side fibers, mathematical statistics and weight statistics were also obtained by their proportions. Energy-dispersive X-ray spectroscopy (EDS) was combined with SEM to analyze the types of elements contained in the fiber material. Fourier transform infrared spectroscopy (FTIR, Thermo Nicolet Corp., Waltham, MA, USA) was employed to determine the position and intensity change of the spectral absorption peak in the wavelength range of 400–4000 cm^−1^. The synthesis, change or disappearance of chemical groups were performed to confirm the formation of SC-PLA. X-ray diffraction (XRD) spectra were characterized by a DX-2700 X-ray diffractometer (Cephas, Taipei, Taiwan) with Cu Kα radiation, whose voltage and current were 40 kV and 40 mA separately. The scanning range was 5–50°, and the scanning speed was 10°/min. Cold crystallization, melting temperature and crystallization of the fiber material were investigated using differential scanning calorimetry (DSC, TA Q2000, TA Instruments, New Castle, DE, USA) in a nitrogen atmosphere to research the thermal property and crystallinity. The test temperature was increased from 25 °C to 300 °C at a heating rate of 10 °C/min, and then was decreased to room temperature at a rate of –10 °C/min. The optical properties of PLLA and SC-PLA crystals were observed by a Polarizing microscope (POM, Carl Zeiss, Jena, Germany).

The water contact angle (WCA) of the fiber membranes was examined by the contact angle goniometer (JY-PHb, Chengde Jinhe Instrument Manufacturing Co., Ltd., Chengde, China) to study the hydrophobicity of the PLA fiber membrane. The pore size distribution was obtained by a pore size tester (TOPAS PSM-165, Frankfurt, Germany) based on the bubbling method. During the measurement, isopropyl alcohol (IPA, Sinopharm Chemical Reagent Co., Ltd., Beijing, China) was dripped to completely infiltrate the sample (the tested area was 2.01 cm^2^). The porosity (P) of the fiber membrane was produced by the following formula [28]:

(1) $$P=\left(1-\frac{\rho }{\rho_{0}}\right)\times 100\%$$

where *ρ*_0_ = 1.27 g/cm^3^, representing the standard density of PLA. Each sample was cut into 5 × 5 cm size, whose thickness and weight were measured with a digital thickness meter (YG141A, Wenzhou Jigao Testing Instrument Co., Ltd., Wenzhou, China) and a balance (FA1004N, Shanghai Jinghai Instrument Co., Ltd., Shanghai, China) to calculate the corresponding density (*ρ*). It was required to select 5 sets of data at different positions to get the average value.

The filtration performance of nanofiber membranes was measured by the filtration test equipment (TOPAS AFC-131, Frankfurt, Germany). The circular tested sample was cut into a diameter of 175 mm and the concentration of NaCl aerosol particles with various diameters was 3 mg/m^3^. The average filtration efficiency and pressure drop of aerosol particles of various diameters under different flow rate conditions were also acquired through testing. According to the formula, the quality factor (QF) was calculated for evaluating the comprehensive filtration performance of the fiber membrane. The air permeability tester (FX 3300, Text test Instrument, Schwerzenbach, Switzerland) was carried out to make a thorough inquiry of the air permeability property under the conditions of a pressure of 200 Pa and a test area of 20 cm^2^.

## 3. Results and Discussion

### 3.1. Morphology and Structure of Side-by-Side Bicomponent Nanofiber Membrane

**Figure 2:**
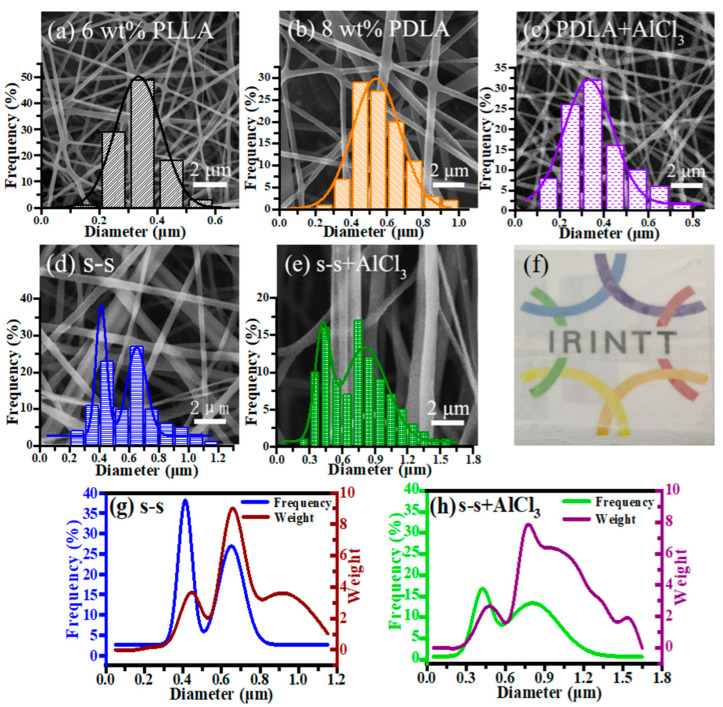
SEM images and nanofiber diameter distributions of (**a**) 6 wt% PLLA, (**b**) 8 wt% PDLA, (**c**) 8 wt% PDLA containing 1 wt% AlCl_3_, (**d**) side-by-side and (**e**) side-by-side containing 1 wt% AlCl_3_ nanofibers; (**f**) the transparency of the side-by-side fibers containing 1 wt% AlCl_3_ nanofibers; number average and weight average distributions of (**g**) side-by-side and (**h**) side-by-side fibers containing 1 wt% AlCl_3_ nanofibers.

A side-by-side electrospinning strategy was employed to produce bicomponent nanofiber in this study. It had been proposed by Tsuji et al. that an electric field was conducive to stretching and increasing the surface area of the molecular chain [29]. PLLA and PDLA spinning solutions were optimized to facilitate the mutual diffusion of the two enantiomeric components’ molecules at the side-by-side contact interface. The single-spinning SEM images of 6 wt% PLLA and 8 wt% PDLA are shown in Figure 2a,b, with the average diameters of 336.1 and 559.9 nm, respectively. In the previous experimental work, we tried LiCl, CaCl_2_, and AlCl_3_ salt additives. Due to the insolubility of high contents of CaCl_2_ and the higher charge density of Li^+^ with smaller atomic radius than Al^3+^, it affected the smooth progress of the electrospinning process [30]. Additionally, excessive tensile force in the electric field made the fiber diameter thinner, which was not conducive to the full diffusion of the molecules on both sides of the bicomponent fiber. Therefore, by introducing different concentrations of AlCl_3_ into 8 wt% PDLA solution, the fiber diameter presented a decreasing and then increasing trend shown in Figure S1, which could be explained by the solution property variation [31,32]. Besides, it could be found in Figure 2c that 8 wt% PDLA with 1 wt% AlCl_3_ had the thinnest diameter of 371.7 nm compared to the fibers with other addition contents, which was comparable to the diameter of 6 wt% PLLA electrospinning nanofiber. It could be clearly illustrated that all single-spinning fibers were uniform without bead defects, and also showed a typical unimodal fiber distribution. Interestingly, each side had an approximately similar diameter, which proved essential to harvest a high proportion of side-by-side nanofibers in our pre-experiments. 

To reduce the electrostatic repulsion effect between two positively charged spinning solutions, a sleeve was set outside the two conjunct tips of needles. The side-by-side fiber (Figure 2d,e) was formed by two fibers arranged tightly in parallel, showing grooves at the intersecting interface [33]. It was concluded that the diameter distribution interval showed an obvious bimodal distribution, representing the diameter concentration area of single fibers and side-by-side fibers separately. As illustrated in Figure 2g,h, both number average and weight average distributions of side-by-side fibers before and after the addition of AlCl_3_ showed consistent double peaks at the small and large diameters. After calculating the integral area under the corresponding curve, compared to the side-by-side fibers without AlCl_3_, the number average had such a drastic improvement from 48.3% to 64.7% statistically, and the weight average increased from 80.3% to 86.4%. These results could be explained by the attraction between Cl^-^ in the PDLA solution and the positive charge in another PLLA solution during the electric field. As a matter of fact, the attraction of the opposite charges would make the two components closer to contact each other, promoting the diffusion of molecules in PLLA and PDLA, which was effective to form side-by-side nanofibers. In particular, it could be further verified from the EDS mapping diagram (Figure S2) that Al elements were uniformly distributed in the prepared s-s+AlCl_3_ nanofiber membrane, indicating that the abundant side-by-side nanofibers were stochastically arranged inside the hierarchical bicomponent nanofiber membrane. Besides, the s-s+AlCl_3_ nanofiber membrane was transparent after adding AlCl_3_ and the fundamental pattern was kept clear, as presented in Figure 2f.

### 3.2. Chemical Structure of Side-by-Side Nanofiber 

**Figure 3:**
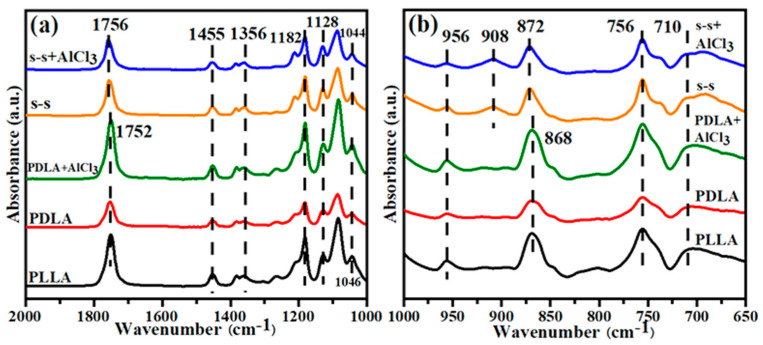
The FTIR spectra of PLLA, PDLA, PDLA+AlCl_3_, s-s, s-s+AlCl_3_ fibers in the range of (**a**) 1000–2000 cm^−1^ and (**b**) 650–1000 cm^−1^.

The chemical structure of single-spinning fiber and side-by-side fiber was characterized by FTIR (Figure 3) in the range of 650–2000 cm^−1^ to confirm the synthesis of the stereo-complex crystalline structure in the bicomponent fiber (the FTIR spectrum of 2000–3500 cm^−1^ is seen in Figure S3). Table 1 recorded in detail the positions of the absorbance peaks corresponding to the stretching and bending vibrations of the related chemical bonds [18,34]. Figure 3a shows the absorbance peak differences of the prepared fibers in the range of 1000–2000 cm^−1^. Compared with single-spinning fibers, the corresponding C=O absorption peak of side-by-side fibers shifted from 1752 to 1756 cm^−1^. Besides, the C–CH_3_ group altered the position from 1046 to 1044 cm^−1^, evidencing the stretching vibrations of the carbonyl group and methyl group under the change of external conditions [35]. It was seen from Figure 3b that the absorbance peak curves of the five samples varied from 650 to 1000 cm^−1^. Moreover, another absorbance peak up-shifted from 868 to 872 cm^−1^, denoting the variation of the C–COO bond, which further illustrated the interaction of the methyl group and carbonyl group to form the hydrogen bond between adjacent PLLA and PDLA molecular chains, driving the formation of SC-PLA [36]. In particular, a significantly new absorbance peak of the side-by-side bicomponent fiber appeared at 908 cm^−1^, demonstrating a new characteristic peak of the stereo-complex crystal with 3_1_ molecular helical conformation [37]. According to the changes in the positions and intensity of the above absorbance peaks, the successful preparation of the side-by-side nanofiber with stereo-complex crystals could be further proved. Furthermore, the FTIR spectra of single-spinning PDLA fiber and side-by-side fiber after the introduction of AlCl_3_ were consistent with before, indicating that the load of AlCl_3_ did not affect the chemical structure of the fibers, and the stereo-complex crystals could be still maintained.

### 3.3. Crystallinity, Crystalline Morphology and Melting Point

**Figure 4:**
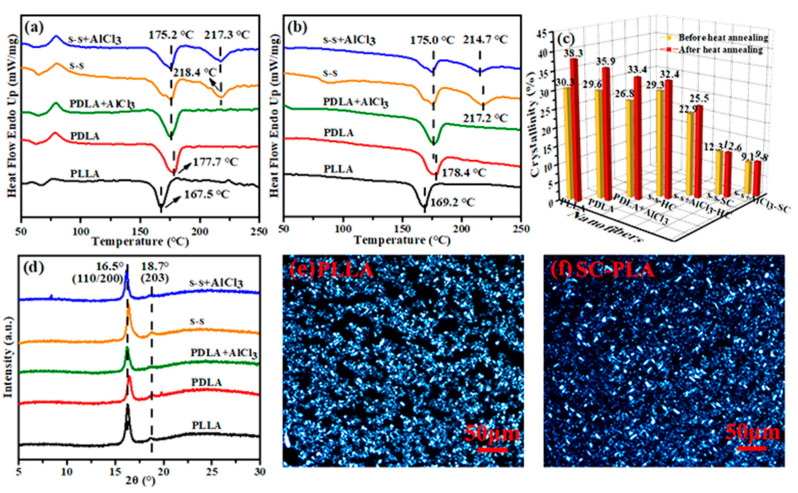
DSC curves of PLLA, PDLA, PDLA+AlCl_3_, s-s, s-s+AlCl_3_ fiber membrane samples (**a**) before and (**b**) after heat annealing at 110 °C; (**c**) the crystallinity data calculated under different conditions; (**d**) XRD curves of five fiber membranes; POM diagrams of (**e**) PLLA and (**f**) SC-PLA.

To verify the chemical structure of the fiber, DSC and XRD characterizations were combined to provide pieces of evidence for the formation of side-by-side nanofibers. Figure 4a,b displays the DSC curves of five nanofiber membrane samples before and after heat annealing at 110 °C, respectively. It could be found that the melting points of PLLA and PDLA in the present study were approximately 168 °C and 178 °C, respectively. The side-by-side fiber maintained a melting peak near 175 °C as well, which was consistent with that of the single-spinning fiber. Interestingly, another melting peak appeared at 217 °C, which was about 50 °C higher than single-spinning PLLA fiber and 40 °C higher than single-spinning PDLA fiber. Two melting peaks at 175 °C and 217 °C indicated that homo-crystals (HC) and stereo-crystals (SC) existed simultaneously, the same as the data in the published literature, which meant the successful synthesis of stereo-complex crystal [38]. Besides, the exothermic peak at about 80 °C (Figure 4a) was the corresponding cold crystallization peak during the heating progress, which disappeared after annealing (Figure 4b) on account of a higher degree of order in the macromolecular chain [39,40,41]. Based on the above DSC curves, the crystallinity (X_c_) could be calculated by the following formula [18]:

(2) $$Xc=\frac{\left|\Delta Hm-\Delta Hcc\right|}{\left|\Delta H_{0}\right|}\times 100{\%}^{\;}$$

where ∆H_m_ and ∆H_0_ are the melting enthalpies of nanofibers and at 100% crystallization, respectively. ∆H_cc_ represents the enthalpy of cold crystallization of nanofibers scanned by DSC in the range of 70–90 °C. As a note, the ∆H_0_ of HC is 93.6 J/g, and the ∆H_0_ of SC is 142 J/g.

Comparing the crystallinity of the samples before and after annealing, it was visually found from Figure 4c that annealing enhanced the crystallinity. This was because heat treatment could promote the mobility of the macromolecular chains in the polymer, so that the amorphous regions were aligned and folded to form crystals [42]. 

Additionally, according to the above formula, the result (Figure 4c) evidenced that the final crystallinity of HC and SC in the s-s+AlCl_3_ fiber membrane after annealing was 25.5% and 9.8%, respectively. Compared with our previous work, the crystallinity was slightly lower. It was illustrated that the raw materials used in this experiment were industrial grade, different from the analytical grade materials before, which led to differences in the optical rotation. Studies have shown that the crystallization performance got worse as the optical rotation decreased [43]. In addition, based on the crystallinity calculated by DSC in the previous work of our research group [18], we also performed a detailed calculation of the ratio of HC to SC as 2:1 in the special sandwich structure, which was higher than the ratio of 3:1 before. Although the overall crystallinity was reduced a bit due to lower optical rotation of industrial raw materials, by introducing AlCl_3_ into PDLA, the SC in the s-s+AlCl_3_ fibers was promoted to a high proportion.

The crystallization performance of five samples was definitively characterized by the XRD test to explore crystallization more comprehensively. As displayed in Figure 4d, five types of fibers were found to have diffraction peaks at 2θ = 16.5° and 18.7°, corresponding to (110/200) and (203) crystal planes of HC with a 10_3_ molecular helical conformation and a pseudo-orthorhombic unit cell structure [35,44]. Unfortunately, the diffraction peak of SC formed by PLLA and PDLA arranged side-by-side in a 3_1_ helical conformation did not appear, which may be attributed to a larger molecular weight of PDLA than that of PLLA used in this experiment. The large molecular weight made the molecular chain severely entangled, restricted its movement ability and increasing the viscosity of the system, thus resulting in insufficient contact between PLLA and PDLA.

In addition, the polarization microscope (POM) was used to observe the crystal morphology, as seen in Figure 4e,f. It was illustrated that the crystal size of SC-PLA was smaller, and over and above that, the number of crystal granules and the nucleation density under the same field of view were also less than that of PLLA, which probably led to insensitive detection of SC by the XRD method. Compared with the previous work, in the case of PLLA and PDLA, unequal mixing in the experiment, excess homo-polymeric chains segments were trapped and dispersed in the sc-crystal, which could reduce the crystallinity and crystallization rate of the sc-crystal and result in stacking into fluffy-structured lamellae in the sc-crystals (Figure 4f) [45]. Meanwhile, after the formation of sc-crystal, it could provide PLLA and PDLA nucleation sites, as a nucleating agent, significantly promoting a homogeneous crystallization process [46,47]. However, based on the SEM, DSC, FTIR, and EDS characteristics, it was proved that SC-PLA was successfully manufactured after the addition of AlCl_3_. The high melting point of the SC was beneficial to improve the heat resistance of the PLA fiber membrane to a certain extent, thus expanding the application range, and having a greater development prospect in the medium- and high-temperature field.

### 3.4. Hydrophobicity and Pore Structure of Fiber Membrane

The wettability of the solid surface is determined by the chemical composition and surface microstructure [48]. The droplets form a composite interface on the fiber surface, where the fibers form a wetting phase, and the air is filled between fibers to form a non-wetting phase [49]. In this regard, Cassie and Baxter proposed an air cushion model, introducing a surface coefficient:

f = fs/(fs + fv)(3)

where fs and fv mean that the droplet is in contact with the solid surface and the air cushion, respectively. 

**Figure 5:**
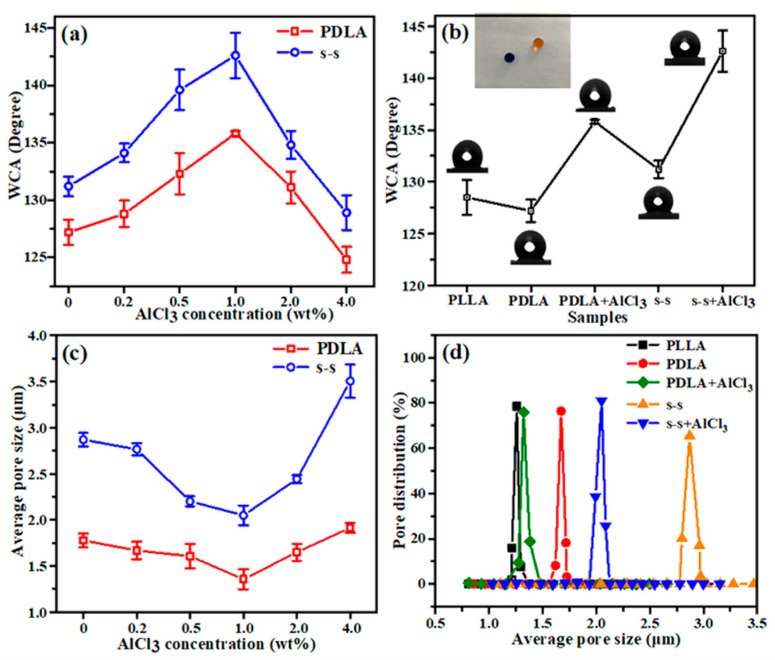
(**a**) Water contact angle (WCA) variation curves and (**c**) the average pore size values of single-spinning PDLA fiber and side-by-side fiber with different contents of AlCl_3_; (**b**) the WCA values and (**d**) the pore size distribution of PLLA, PDLA, PDLA+AlCl_3_, s-s, s-s+AlCl_3_ fiber membrane samples.

The contact angle can be calculated by the following equation:

cosθ’ = f cosθ + f − 1(4)

which shows that water contact angle (WCA) will increase with the decrease in value f [50]. The WCA of different samples is shown in Figure 5a,b. As seen in Figure 5a, it expressed the same variety trend of increasing first and then decreasing on the WCA for single-spinning and side-by-side fiber with different contents of AlCl_3_. Importantly, the fibers with small diameters constructed high porosity, which expanded the contact area between liquid and air, leading to a decrease in f value and further enhancing the hydrophobicity of the fiber membrane. In addition, it was found that all the WCA of the side-by-side fibers was larger than that of the single-spinning fiber with the same salt addition, which could be explained by the fact that the groove structure on the surface of the side-by-side fibers and the stochastic structure among single and side-by-side fibers increased the integrated roughness in the membrane. Figure 5b conveys the WCA of the five fiber membranes, showing that the WCA of the side-by-side fiber with 1 wt% AlCl_3_ reached 142.9°, which was relatively close to the superhydrophobic criteria (150°), and thus the membrane was conducive to antibacterial, anti-fouling, self-cleaning properties for practical applications.

It is known that the size and distribution of the fiber diameter, the thickness of the fiber membrane, and the spinning state undoubtedly affect the pore size of the nanofiber membrane. Accordingly, the samples investigated in the pore size tests needed to maintain the invariable spinning conditions, most importantly, the thickness must be equal. It is presented in Figure 5c that as salts are added progressively, the average pore size of the fiber shows a decreased and then increased tendency, which corresponds to the change in diameter. By the continuous rotation of the roller receiver device during the electrostatic spinning process, the fine nanofibers were collected layer by layer, generating a more compact arrangement structure, and consequently, the average pore size of the fiber membrane was reduced. While the content of AlCl_3_ exceeded 1 wt%, the fibers transformed a thicker diameter and the average pore size increased correspondingly. The single-spinning fiber and side-by-side fiber containing 1 wt% AlCl_3_ had the smallest average pore size of 1.32 and 2.05 μm respectively. Additionally, the pore size distribution was also concentrated with 1 wt% AlCl_3_ incorporated, as presented in Figure 5d. For the filter material, the size and distribution of the pore size greatly impact the filtration efficiency. The small pore size is favorable for the screening of aerosol particles in the filtration process in order to optimize the interception effect and filtration performance. Hence, the selection of 1 wt% additive might be the most beneficial for the improvement of filtration performance.

The average diameter, thickness, average pore size, and porosity data of five fiber membranes are presented in detail in Table 2. It was evidently found that the average pore size and porosity of the single-spinning and side-by-side fibers with 1 wt% AlCl_3_ were smaller and higher than no additive, respectively, which could be explained by the reason that thin fibers had a small average pore size, large specific surface area and a large number of microscopic voids existing between the fibers, advantageous to the majorization of filtration performance. Because of the combination of single and side-by-side nanofibers, the average pore size of the side-by-side fiber membrane was inevitably larger than that of the single-spinning PLLA and PDLA fibers. However, thanks to the well-organized pore size distribution and three-dimensional tortuous microstructure, a side-by-side nanofiber membrane with 1 wt% AlCl_3_ could bring about an unexpected improvement of filtration performance.

### 3.5. Filtration Performance of Fiber Membrane

**Figure 6:**
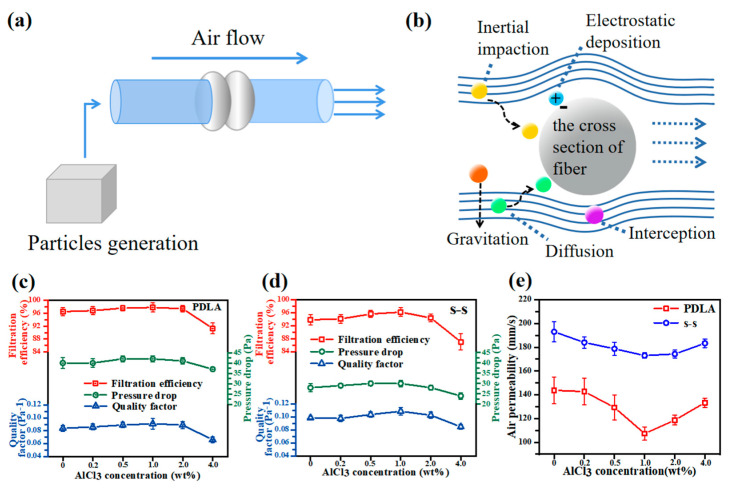
(**a**) The schematic diagram of filtration equipment; (**b**) the filtration mechanism diagram of nanofiber filter materials; the filtration efficiency, pressure drop, and quality factor of (**c**) single-spinning PDLA fibers and (**d**) side-by-side fibers with different AlCl_3_ contents; (**e**) the air permeability performance of single-spinning PDLA fibers and side-by-side fibers with different AlCl_3_ contents.

Most bacteria, viruses and other microorganisms are transmitted through aerosol particles, illustrating the importance of developing a filter with excellent filtration performance. Here, the equipment shown in Figure 6a was used to test the filtration efficiency and pressure drop of each fiber membrane sample. As presented in Figure 6b, the filtration process of nanofiber filter materials for aerosol particles was a synergetic effect of inertial impaction, interception, diffusion, electrostatic adsorption, and gravitation mechanisms [7,51]. The filtration efficiency, pressure drop and air permeability of single-spinning fibers and side-by-side fibers under different AlCl_3_ content conditions were evaluated in detail (Figure 6c–e) so as to analyze the effect of AlCl_3_ particles on the filtration performance. It was seen that the variation curves of filtration efficiency and pressure drop were displayed similarly, presenting an increase prior to a decreasing trend, which could be explained as consistent with the trend of average diameter and average pore size. A small pore size could provide such a tight structure that the possibility of the collision between the filter fiber and aerosol particles contaminant became higher. As a result, particles were easy to be captured, bringing out a higher filtration efficiency due to the combined action of several mechanisms. However, high filtration efficiency generally accompanied the challenge that the airflow was subject to higher resistance while passing, inducing a high pressure drop and a shortened service life. Over and above, the air permeability data (Figure 6e) further demonstrated this phenomenon. According to the values of filtration efficiency and pressure drop, the corresponding QF value could be calculated using the formula:

(5) $$QF=-\frac{Ln\left(1-\eta \right)}{\Delta P}$$

where η represents the filtration efficiency, ΔP expresses the pressure drop [52]. As the content of AlCl_3_ was 1 wt%, the QF of side-by-side fibers was the highest, at 0.109 Pa ^−1^, which was an increase of 19.8% compared to the single-spinning fibers with the same content, indicating that the comprehensive filtration performance of the nanofiber membrane was the best under this additive concentration.

**Figure 7:**
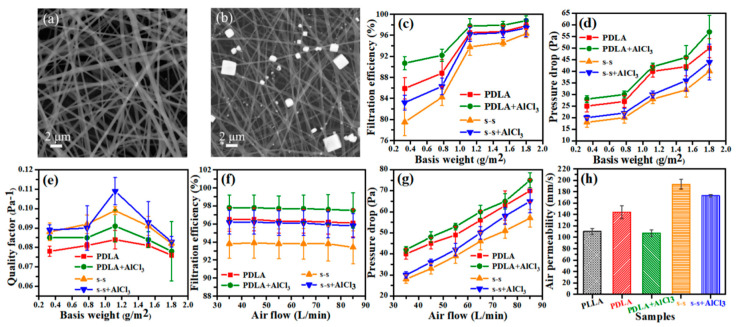
SEM images of the s-s+AlCl_3_ nanofibers (**a**) before and (**b**) after filtration; (**c**) the filtration efficiency, (**d**) pressure drop, and (**e**) quality factor of single-spinning and side-by-side fibers before and after adding 1 wt% AlCl_3_ under different basis weight conditions; (**f**) the filtration efficiency and (**g**) pressure drop of single-spinning and side-by-side fibers before and after adding 1 wt% AlCl_3_ under different airflow conditions; (**h**) the air permeability performance of PLLA, PDLA, PDLA+AlCl_3_, s-s, s-s+AlCl_3_ fiber membranes.

As shown in Figure 7a,b, SEM images illustrated the changes before and after filtration, evidently manifesting that NaCl particles of different sizes were successfully intercepted in the interstices of fibers after filtering. It is known that the basis weight of the fiber membrane is closely related to the thickness, which is negatively related to the air permeability. Therefore, by adjusting the basis weight under different application situations, a balance between high filtration efficiency and low-pressure drop can be fulfilled. It could be evidenced that the continuing rise of the basis weight was conducive to increasing the filtration efficiency and pressure drop (Figure 7c,d) because of the increased density of nanofibers. Especially when the basis weight varied from 0.3 to 1.1 g/m^2^, the filtration efficiency was enormously enhanced, which could reach 94–98%. In the meantime, the pressure drop did not change a lot, merely increasing by less than 15 Pa. In contrast, as the basis weight was mounted to 1.5 and 1.8 g/m^2^, the alteration in filtration efficiency was not distinct, while the pressure drop increased sharply. By virtue of high efficiency and low pressure drop, the 1.1 g/m^2^ side-by-side fiber membrane with 1 wt% AlCl_3_ addition had the best comprehensive filtration performance, and the calculated QF value was 0.109 Pa^−1^ as shown in Figure 7e. The diameter distribution of as-prepared side-by-side fibers was wide, including thick bicomponent fibers in parallel and fine single fibers separated, which was similar to the tree-like nanofibers [53]. In this structure, the thick fiber served as the frame of the fiber membrane, playing a supporting role and the fine fiber was embedded in it, as a connecting tool, increasing the specific surface area and reducing the pore size. After the fibers were continuously deposited and stacked together, a fiber membrane with a unique structure that could better intercept small-diameter aerosol particles was formed, not only efficiently filtering particles of diverse particle sizes (Figure S4), but also obviously cutting down pressure drop. Besides, adequate air permeability made the fiber membrane complete brilliant filtration performance perfectly without sacrificing filtration efficiency.

Additionally, it is demonstrated in Figure 7f,g that the impression of different airflow rates on filtration efficiency and pressure drop under the condition of 1.1 g/m^2^ basis weight. As the airflow rate rose from 35 to 85 L/min, the filtration efficiency did not vary tremendously, while when the pressure drops gradually it expressed a linear upward trend. It is worth noting that the fiber membrane still maintained a valuable interception effect on aerosol particles and stable filtration efficiency, expanding the application range under various airflow rates. Figure 7h summarizes the air permeability data of the five fabricated samples, further confirming that the side-by-side fiber membrane had better air permeability. In addition, it was found from Figure S5 that compared to the other three samples, our fabricated membrane showed superiority. In short, the side-by-side fiber membrane with 1 wt% AlCl_3_ can be applied in filtration, separation, biomedicine and other fields owing to its high efficiency, low pressure drop, and superior air permeability.

## 4. Conclusions

In summary, we have prepared a continuous and uniform PLA side-by-side bicomponent fibrous membrane containing salt particles through the side-by-side electrospinning technology. Benefiting from the properties of the PLA material, the influence of AlCl_3_ salt on the fibers, and the special structure of the coexistence of thick and thin fibers, the resulting nanofiber membrane held excellent hydrophobicity, outstanding filtration efficiency, low pressure drop, and superior air permeability. The addition of AlCl_3_ within a certain range reduced the fiber diameter and pore size while it increased the specific surface area and porosity, which were all in favor of enhancing the hydrophobicity and the filtration performance of the side-by-side fiber membrane. Especially when the basis weight was 1.1 g/m^2^, the filtration efficiency reached 96.2%, while the pressure drop was only 30 Pa, and the air permeability attained 173 mm/s. As a result, the QF value was 0.109 Pa^−1^, which was much higher than the common single-component fibers, exhibiting better comprehensive filtration performance. This work provides a hopeful candidate for the effective filtration of particulate pollutants and the useful prevention of the spread of bacteria, viruses, and other microorganisms in the air, so that it can be employed in filtration, separation, biomedicine, personal protection and other fields.

## Figures and Tables

**Table 1:** The chemical bond vibrations were detected from the FTIR spectra for PLA nanofibers.

Assignment	Wavenumber (cm^−1^)	Assignment	Wavenumber (cm^−1^)
Stretching of C=O	1752–1756	Rocking of CH_3_	956
Asymmetric bending of CH_3_	1455	The characteristic diffraction peak of SC-PLA	908
Bending of CH	1356	Stretching of C–COO	868–872
Stretching of C=O	1128	Stretching of C–H	756
Stretching of –C–CH_3_	1046–1044	Stretching and bending of C–H	710

**Table 2:** The average diameter, thickness, average pore size and porosity data of five fiber membrane samples.

Sample	PLLA	PDLA	PDLA + AlCl_3_	s-s	s-s + AlCl_3_
Average diameter (μm)	0.3360 ± 0.022	0.5599 ± 0.016	0.3717 ± 0.008		
Thickness (μm)	25 ± 5	26 ± 9	29 ± 6	25 ± 7	29 ± 7
Average pore size (μm)	1.26 ± 0.223	1.67 ± 0.180	1.32 ± 0.181	2.87 ± 0.178	2.05 ± 0.175
Porosity (%)	94.2 ± 1.3	93.4 ± 1.2	95.0 ± 0.8	87.7 ± 1.4	88.5 ± 1.2

## Data Availability

Not applicable.

## Supplementary Materials

The following are available online at https://www.mdpi.com/article/10.3390/membranes11110819/s1, Figure S1: SEM images and diameter values statistics of single-spinning PDLA fibers with varied contents of AlCl_3_. Figure S2: EDS mapping diagram of the side-by-side fiber with AlCl_3_. Figure S3: The FTIR spectra of PLLA, PDLA, PDLA+AlCl_3_, s-s, s-s+AlCl_3_ fibers in the range of (a) 2000–3500 cm^−1^, (b) 1000–2000 cm^−1^ and (c) 650–1000 cm^−1^. Figure S4: The filtration efficiency of prepared PDLA, PDLA+AlCl_3_, s-s, s-s+AlCl_3_ fiber membranes of NaCl aerosol particles with different sizes. Figure S5: (a) The filtration efficiency, pressure drop, and (b) QF value of meltblown PE fiber-800 μm, PE/PA fiber-4.5 μm, nylon electrospinning fiber and our prepared s-s+AlCl_3_ side-by-side bicomponent fiber.
