# Supplementary material for: Bicomponent PLA Nanofiber Nonwovens as Highly Efficient Filtration Media for Particulate Pollutants and Pathogens
Source: Membranes (Basel). 2021 Oct 27;11(11):819. doi: 10.3390/membranes11110819 (PMC8622781; doi:10.3390/membranes11110819)
Supplement: Supplementary file 1 [file membranes-11-00819-s001.zip › membranes-1432077-supplementary.pdf]

membranes

Supplementary Material

Bicomponent PLA Nanofiber Nonwovens as Highly Efficient Filtration Media for Particulate Pollutants and Pathogens

Danyang Gao[1], Renhai Zhao[1,*], Xue Yang[1], Fuxing Chen[1,*], Xin Ning[1,*]

1 Industrial Research Institute of Nonwovens & Technical Textiles, College of Textiles & Clothing, Shandong Center for Engineered Nonwovens, Qingdao University, Qingdao 266071, China; gdykdy@163.com (D.G.); 2017021352@qdu.edu.cn (X.Y.)

* Correspondence: chinesezrh@126.com (R.Z.); fuxing1991@gmail.com (F.C.); xning@qdu.edu.cn (X.N.); Tel.: +86-532-85953572 (X.N.).

The Effect of $AlCl_3$ Addition on the Diameter of Nanofibers

The addition of salts was used frequently to increase the conductivity, promote electrospinning, and improve the morphology of fibers. However, it was considered to select an appropriate content of addition. When the content of AlCl3 increased to 6.0 wt%, the solution converted turbid, indicating that the solubility of AlCl3 had been exceeded. By doing so, the content of AlCl3 was linearly increased to 4.0 wt% for the aim of investigating the effect of multiple addition contents on the morphology of the nanofibers (Figure S1 a-e) in this experiment. The histogram (Figure S1 f) diametrically presented the change in diameter caused by multifarious contents. With the increase of the concentration of AlCl3 inorganic salt until 1.0 wt%, the fiber diameter gradually became thinner. This was attributed to high conductivity of the polymer solution causing greater electric field force the jet received under the high-voltage electric field during the spinning process, which was advantageous to the stretch of the fiber, certainly, the fiber diameter decreased accordingly. Whereas as the AlCl3 salt was more introduced, the fiber diameter got thicker instead. It might be that the excessive introduction of salts led to the formation of a high-viscosity solution, which restricted the movement of the molecular chain, resulting in the weakening of the ability of the jet to be stretched by the electric field at the same voltage, making the fiber thicker1, 2. In comparison, inserting 1.0 wt% AlCl3 was the most suitable quantity to possess thinner and more uniform fibers. At the same time, it could be further verified from the EDS mapping diagram (Figure S2) of the side-by-side fiber with AlCl3. It was found that in addition to the three elements C, H, and O contained in PLA itself, there was also Al element simultaneously, indicating that AlCl3 was uniformly distributed in the prepared fiber membrane.

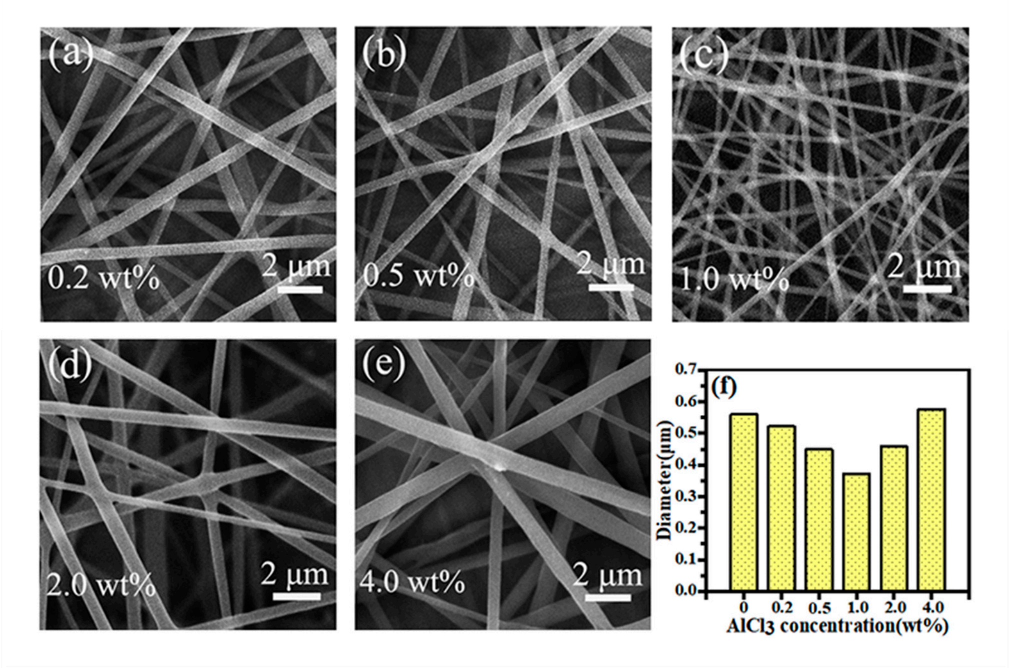

Figure S1. SEM images and diameter values statistics of single-spinning PDLA fibers with varied contents of $AlCl_3$.

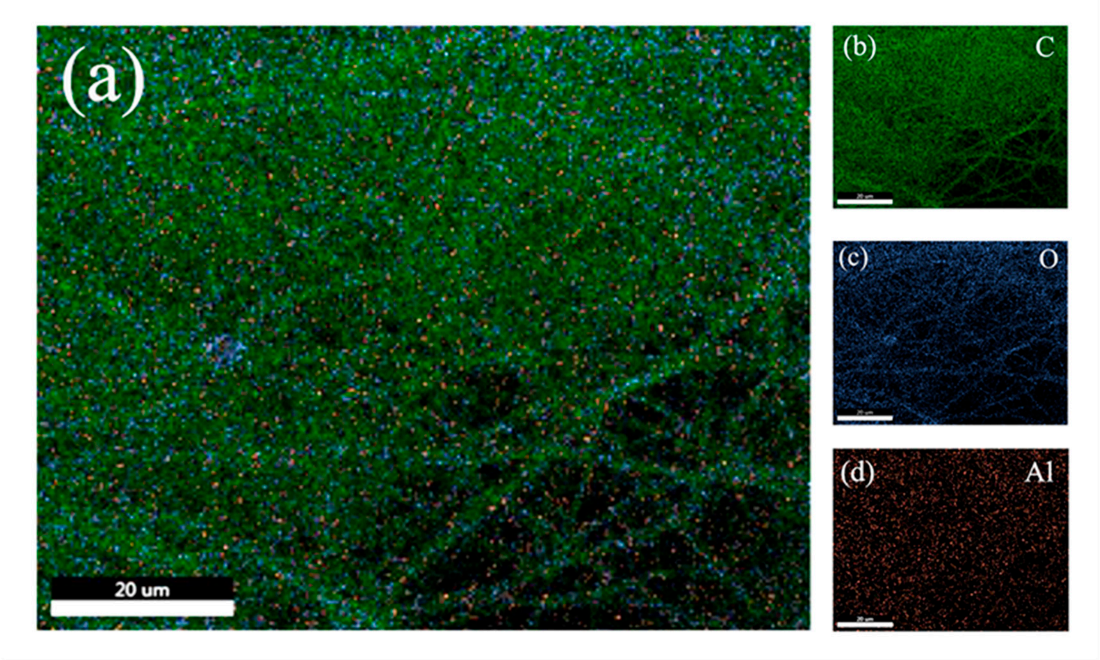

Figure S2. EDS mapping diagram of the side-by-side fiber with $AlCl_3$.

FTIR Spectrum from 650-3500 cm^{-1}

It was seen from Figure S3 that FTIR spectra were detected from 650-3500 cm^{-1}. The absorbance corresponding to the vibrations of each chemical bond was listed in Table 1. In addition, what needed to be added here was that the asymmetric stretching vibration of CH_3 appeared at 2996 and 2945 cm^{-1}, and the stretching vibration of CH appeared at 2880 cm^{-1}, further indicating the chemical structure of PLA [3].

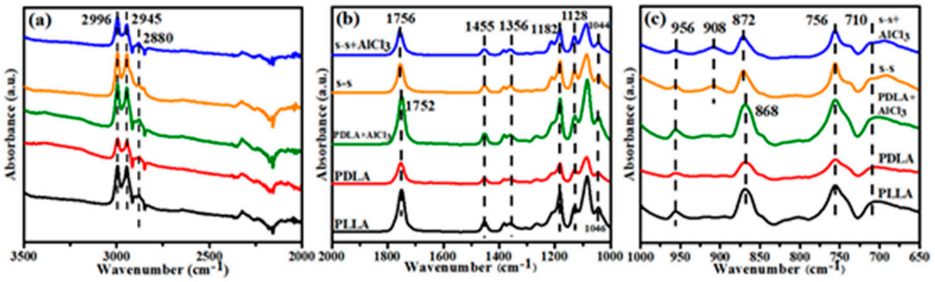

Figure S3. The FTIR spectra of PLLA, PDLA, PDLA+$AlCl_3$, s-s, s-s+$AlCl_3$ fibers in the range of (a) 2000-3500 cm^{-1}, (b) 1000-2000 cm^{-1} and (c) 650-1000 cm^{-1}.

The Ratio of HC and SC Calculated in the Sandwich Structure

Based on the crystallinity calculated by the DSC characterization of the annealed sample (Figure 4b and c), here we carried out a detailed calculation of the ratio of HC to SC in the s-s+$AlCl_3$ fiber membrane. The crystallinity of HC in the single-spinning PLLA and PDLA fibers were 38.3% and 33.4%, respectively. Additionally, the HC and SC crystallinity in the s-s+$AlCl_3$ fiber reached about 25.5% and 9.8%, separately. We needed to subtract the contribution of the single fibers in the bicomponent fiber membrane to HC to obtain its true crystallinity. Assuming that the crystallinity of the split single fibers in the bicomponent fiber membrane was the same as the single-spinning PLLA and PDLA samples, which was calculated as the average crystallinity of 35.85%. Therefore the split single fibers with the weight average statistics of about 15% would contribute 5.4% of the HC in the bicomponent sample, which also meant that the outer areas of the bicomponent fiber contributed 20.1% of the HC. Thus, the ratio of HC crystals and SC crystals in the sandwich structure of the bicomponent fiber achieved about 2:1, which was a considerable improvement compared to our previous work.

The Filtration Efficiency of NaCl Aerosol Particles of Different Sizes

In this experiment, we also tested the filtration efficiency of the prepared membranes on NaCl aerosol particles with different sizes. As shown in Figure S4, as the aerosol particle size increased, the filtration efficiency also gradually increased, indicating that the fiber membrane was easier to intercept large particles. In particular, when the particle size was 0.225 μm, the filtration efficiency of the s-s+$AlCl_3$ fiber membrane had reached 95%, which was of great significance for the filtration of small particles.

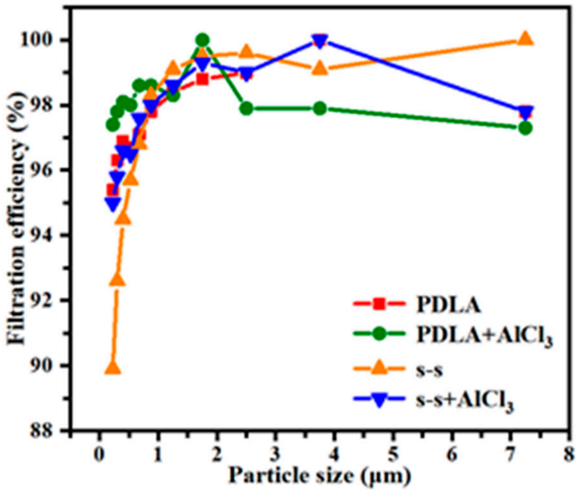

Figure S4. The filtration efficiency of prepared PDLA, PDLA+$AlCl_3$, s-s, s-s+$AlCl_3$ fiber membranes of NaCl aerosol particles with different sizes.

The Filtration Effect Comparison of Prepared s-s+$AlCl_3$ Bicomponent Membrane

Here we compared our bicomponent membrane with some samples in the published literature to explore the filtration performance[4, 5]. As shown in Figure S5, it was found that the filtration efficiency of meltblown PE fiber membrane was significantly lower than that of electrospinning fibers, which was caused by its large pore size. Besides, electrospinning PA fibers were entangled on the meltblown fibers as the bottom layer, the filtration efficiency was greatly improved, but at the same time, the pressure drop also increased dramatically, which reflected the imbalance between high filtration efficiency and pressure drop. In contrast, our sample had a filtration efficiency of up to 96.2% and a pressure drop of 30 Pa, with bigger QF values as 0.109 Pa^{-1}, indicating the excellent comprehensive filtration performance, which meant a promising development prospect.

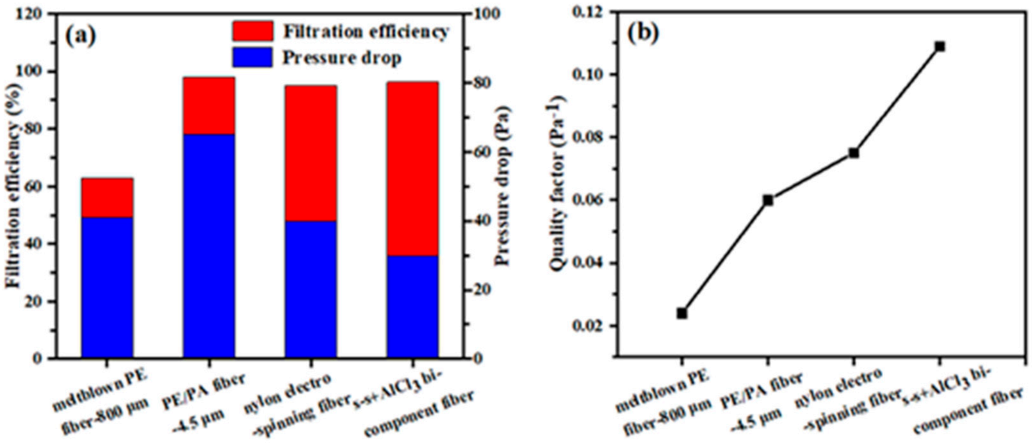

Figure S5. (a) The filtration efficiency, pressure drop, and (b) QF value of meltblown PE fiber-800 μm, PE/PA fiber-4.5 μm, nylon electrospinning fiber and our prepared s-s+$AlCl_3$ side-by-side bicomponent fiber.

References

1. Soberman, M. J.; Farnood, R. R.; Tabe, S., Functionalized powdered activated carbon electrospun nanofiber membranes for adsorption of micropollutants. *Separation and Purification Technology* **2020,** *253*, 10.

2. Singh, Y. P.; Dasgupta, S.; Nayar, S.; Bhaskar, R., Optimization of electrospinning process & parameters for producing defect-free chitosan/polyethylene oxide nanofibers for bone tissue engineering. *J. Biomater. Sci.-Polym. Ed.* **2020,** *31* (6), 781-803.

3. Pan, P. J.; Yang, J. J.; Shan, G. R.; Bao, Y. Z.; Weng, Z. X.; Cao, A.; Yazawa, K.; Inoue, Y., Temperature-Variable FTIR and Solid-State C-13 NMR Investigations on Crystalline Structure and Molecular Dynamics of Polymorphic Poly(L-lactide) and Poly(L-lactide)/Poly(D-lactide) Stereocomplex. *Macromolecules* **2012,** *45* (1), 189-197.

4. Xu, Y. Q.; Zhang, X. M.; Hao, X. B.; Teng, D. F.; Zhao, T. N.; Zeng, Y. C., Micro/nanofibrous nonwovens with high filtration performance and radiative heat dissipation property for personal protective face mask. *Chemical Engineering Journal* **2021,** *423*, 9.

5. Niu, Z. L.; Bian, Y.; Xia, T. L.; Zhang, L.; Chen, C., An optimization approach for fabricating electrospun nanofiber air filters with minimized pressure drop for indoor PM2.5 control. *Build. Environ.* **2021,** *188*, 11.
